# Maternal body mass index is not associated with assisted reproductive technology outcomes

**DOI:** 10.1038/s41598-023-41780-4

**Published:** 2023-09-08

**Authors:** Nobuyuki Kidera, Tomonori Ishikawa, Toshihiro Kawamura, Naoyuki Miyasaka

**Affiliations:** 1 Denentoshi Ladies Clinic, 1-5-1 Azamino Aoba-ku, Yokohama-shi, Kanagawa-ken 225-0011 Japan; 2 Department of Comprehensive Reproductive Medicine, Tokyo Medical and Dental University, 1-5-45 Yushima, Bunkyo-ku, Tokyo-to 113-8510 Japan; 3 Department of Perinatal and Maternal Medicine (Ibaraki), Tokyo Medical and Dental University, 1-5-45 Yushima, Bunkyo-ku, Tokyo-to 113-8510 Japan

**Keywords:** Medical research, Risk factors

## Abstract

The effects of body mass index (BMI) on assisted reproductive technology (ART) outcomes such as ovarian dysfunction, poor ovum quality, and endometrial dysfunction have been studied; however, many aspects remain controversial. Therefore, we retrospectively investigated the relationship between BMI and ART outcomes. For 14,605 oocyte retrieval cycles at our hospital between January 2016 and December 2020, BMI was divided into five groups (< 18.5, 18.5–20.0, 20.0–22.5, 22.5–25.0, ≥ 25 kg/m^2^) and measured before oocyte retrieval. The normal fertilization and high-grade blastocyst rates were compared. In addition, in the 7,122 frozen-thawed embryo transfers (FET) with highest-grade embryos, the clinical pregnancy, miscarriage, and live birth rates were investigated in the five BMI groups. Multiple regression analysis on normal fertilization and high-grade blastocyst rates revealed no statistically significant differences. Furthermore, after propensity score matching on FET, there was no significant difference in clinical pregnancy, miscarriage, and live birth rates in the BMI groups. BMI is a risk factor for complications during pregnancy; however, it does not affect ART outcomes. Therefore, we believe weight guidance should be provided to women with obesity at the start of infertility treatment, but treatment should not be delayed.

Obesity is reportedly associated with ovarian dysfunction, poor response to ovarian stimulation, poor ovum quality, endometrial dysfunction, and low birth rate in women. However, recent reports have contradicted this, making the effect of body mass index (BMI) on assisted reproductive technology (ART) outcomes controversial. In addition, the BMIs of East Asian and Southeast Asian women, such as those in Japan, are relatively lower than those of Western women ^1^, but there are few reports on the effects of low BMI.

To emphasize the controversy, in a previous report on ART, BMI was associated with embryogenesis ^2^; however, no effect was reported ^3^. One previous study reported no difference in the ratio of chromosomal aneuploidy between women with and without obesity in preimplantation genetic testing for aneuploidy ^4,5^. In addition, obesity in women has been associated with decreased pregnancy or live birth rates ^6–11^, although some reports suggest no such association ^2,12–14^. There have been reports of increased miscarriage rates in women with obesity ^6,7,15–17^; however, other studies report no such difference ^12,14,18^. Therefore, the effect of BMI on ART outcomes remains unclear. In addition, the effect of low BMI, such as East Asians, is also unclear. One study reported that pregnancy rates were lower in women with low body weight than in those with normal body weight, but could not explain the effects of obesity. Further, previous reports divided BMI into three or four groups based on the World Health Organization guidelines; however, this classification has not given clear results.

In this study, we aimed to clarify the optimal BMI for ART in Japan by analyzing ART outcomes, investigating the effect of BMI on ART by subdividing BMI more finely than in previous reports, and performing a multivariate analysis to exclude multiple confounding factors.

## Materials and methods

Following the World Health Organization guidelines, we divided BMI into five groups (< 18.5, 18.5–20.0, 20.0–22.5, 22.5–25.0, and ≥ 25.0 kg/m^2^). The normal fertilization and high-quality blastocyst (Gardner Schoolcraft criteria of ≥ 3BB) ^19^ rates among the BMI groups in 14,605 oocyte retrieval cycles performed at our hospital between January 2016 and December 2020 were investigated. BMI was calculated by dividing the weight in kilograms by the square of the height in meters (kg/m^2^).

Among the BMI groups mentioned above, we investigated the 7,122 cycles with the highest-grade embryo (Gardner Schoolcraft criteria of ≥ 3AA) in the 13,301 cycles of frozen-thawed embryo transfer (FET) at our hospital during the same period. Maternal weight was measured before oocyte retrieval and FET. Furthermore, maternal height was reported in the questionnaire and used to calculate BMI.

The outcome measures were the normal fertilization and high-quality blastocyst rates in oocyte retrieval, clinical pregnancy, miscarriage, and live birth rate in FET. In this study, ultrasound confirmation of the presence of the gestational sac was defined as clinical pregnancy; stillbirths after 22 weeks of gestational age were considered miscarriages.

All procedures were conducted in compliance with the ethical standards of the responsible committee on human experimentation (institutional and national), the 1964 Helsinki Declaration, and its later amendments. The Clinical Ethics Committee of Denentoshi Ladies Clinic approved this retrospective study (20071604). Informed consent was obtained from all the participants.

### ART procedure and clinical pregnancy definition

Ovarian stimulation was performed on the 3rd day of menstruation. For this, clomiphene citrate was prescribed at 50 mg/day and used with recombinant follicle-stimulating hormone and human menopausal gonadotropin unless the antral follicle count and anti-Mullerian hormone levels showed poor ovarian reserve. Clomiphene citrate alone was administered when the ovarian reserve was low. Furthermore, oocyte retrieval was performed 35 to 37 h after administering recombinant human chorionic gonadotropin hormone (Ovidrel, EMD Serono, Switzerland) or gonadotropin-releasing hormone agonist (Buserecur, Fuji Parma, Japan) when one or more ovarian follicles reached ≥ 18 mm in diameter on ultrasound examination. Spermatozoa were obtained using density gradient centrifugation, and a universal in vitro fertilization (IVF) medium with phenol red (Origio, Japan) was used as the fertilization medium. The insemination concentration was set at 1.0–1.5 × 10^5^ /ml. After dropping the sample into the Makler chamber, spermatozoa were analyzed using a sperm motility analysis system (SMAS, DITECT, Japan). Embryos were exposed to a global total medium (Astec, Japan). Intracytoplasmic sperm injection (ICSI) was performed using standard techniques.

Fertilization was evaluated 18–19 h after IVF or ICSI; it was considered normal when two distinct pronuclei were evident. Embryos were assessed using a stereoscope (Olympus SZX16; Olympus Corporation, Shinjuku, Japan), and the following parameters were recorded: morphological classification (Gardner Schoolcraft criteria) of the inner cell mass, trophectoderm morphology, and cavity expansion.

Cryopreservation of embryos was performed using a vitrification kit. In addition, FET was performed using the hormone replacement cycle or natural cycle. The transfer date for blastocysts was 5 days after ovulation or 5 days after the start of luteal support. In contrast, the transfer date for the cleavage stage was 3 days after ovulation or 3 days after the start of luteal support. In the hormone replacement cycle FET, estradiol administration usually starts on the 3rd day of the menstrual period. When the endometrial thickness was ≥ 7 mm on the 11th to 19th day of the menstrual period, luteal support was initiated and continued until the 9th week of gestation. In the natural cycle FET, a progesterone concentration of ≥ 1.00 ng/mL was defined as ovulation. From the 3rd day after ovulation to the 4th week of gestation, a progesterone vaginal suppository was used. A thawing medium (KITAZATO BioPharma, Japan) was used for embryo thawing. If an appropriately increasing serum human chorionic gonadotrophin level was encountered, transvaginal ultrasound was performed at 5–6 weeks of gestation to determine the presence of a gestational sac, indicating clinical pregnancy. Based on questionnaires obtained for all patients, the delivery procedure, complications, gestational age, infant's weight, and sex were investigated.

### Statistical analysis

Multiple regression analysis was used to calculate the standardized partial regression coefficient (β) and P-value by adjusting for maternal age, first cycle in oocyte retrieval, history of delivery or miscarriage, endometriosis, male factor infertility, and anti-Mullerian hormone to investigate the effect of BMI on normal fertilization rate and high-grade blastocyst rate. Cases with sperm parameters less than the WHO 2010 definition (volume 1.5 mL, total sperm number 39 million per ejaculate, sperm concentration 15 million per mL, total motility 40%, morphologically normal forms 4.0%) were considered male factor infertility ^20^. The nominal scale was used as a dummy variable. We also confirmed that all the variance inflation factors were < 10 for multicollinearity. Residuals and outliers were examined using the Durbin–Watson test. In addition, a multivariate logistic regression model was used to calculate the adjusted odds ratio (OR) with a 95% confidence interval (CI) for the effect of BMI on pregnancy outcomes in FET with propensity score (PS) matching. The PS model was estimated using a logistic regression model adjusted for potential confounding covariates such as maternal age, first cycle in oocyte FET, history of delivery, history of miscarriage, double embryo transfer, day 5 blastocyst transfer, and uterine factor infertility. The PS was matched using the nearest-neighbor algorithm with a caliper width of 0.2 logits of the standard deviation. The *P*-value was calculated using chi-square tests among five BMI groups for clinical pregnancy, delivery, and miscarriage. If there were significant differences, residual analysis was then performed.

Confounding factors were selected based on previous studies ^8,15^. Difference*s* between the five BMI groups for the population characteristics in oocyte retrieval and FET were calculated using chi-square tests for categorical variables and Kruskal–Wallis tests for maternal age.

Statistical analysis was performed using the SPSS version 26.0 software (SPSS, Inc., Chicago, IL, USA). To evaluate the normal distribution, the Kolmogorov–Smirnov test was used. The results are expressed as the median, interquartile range (IQR), 95% CI, and *P*-values. Statistical significance was set at *P* < 0.05 for all tests.

## Results

The characteristics of the confounding factors in oocyte retrieval are shown in Table 1. Multiple regression analysis of IVF showed that only male factor infertility was associated with a normal fertilization rate. Meanwhile, maternal age and the first cycle in oocyte retrieval were associated with a high-grade blastocyst rate. BMI was not associated with normal fertilization or high-grade blastocyst rate (Table 2). Intracytoplasmic sperm injection (ICSI), maternal age, first cycle in oocyte retrieval, and delivery history were associated with the normal fertilization rate. Maternal age and the first cycle in oocyte retrieval were also associated with the high-grade blastocyst rate. The univariate analysis of ICSI showed significant differences in normal fertilization and high-grade blastocyst rates with increasing maternal BMI. However, after controlling for significant confounders, BMI did not significantly predict normal fertilization or high-grade blastocyst rates (Table 2).

**Table 1:** Population characteristics in oocyte retrieval.

	Body Mass Index (kg/m^2^)					
	< 18.5	18.5–20.0	20.0–22.5	22.5–25.0	≥ 25.0	

	n = 2039	n = 3654	n = 5235	n = 2476	n = 1201	*P*-values^a^
Maternal age, year, median	38.0	39.0	40.0	40.0	41.0	< 0.001
(IQR)	(31.0–45.0)	(33.0–45.0)	(34.0–46.0)	(34.0–46.0)	(37.0–45.0)	
First cycle in oocyte retrieval (%)	979 (48.0)	1536 (42.0)	2010 (38.4)	831 (33.5)	362 (30.1)	
History of delivery (%)	2039 (27.3)	961 (26.3)	1324 (25.2)	586 (23.6)	223 (18.5)	< 0.001
History of miscarriage (%)						
≥ 1	506 (24.8)	1168 (31.9)	1699 (32.4)	868 (35.0)	365 (30.3)	< 0.001
≥ 2	114 (5.5)	365 (9.9)	430 (8.2)	213 (8.6)	107 (8.9)	< 0.001
Endometriosis (%)	158 (7.7)	287 (7.8)	303 (5.7)	117 (4.7)	83 (6.9)	< 0.001
Male factor infertility^b^	1168 (57.2)	2195 (60.0)	3253 (62.1)	1565 (63.2)	813 (67.6)	< 0.001
AMH, ng/ml, median	1.6	1.4	1.3	1.2	1.0	< 0.001
(IQR)	(0.1–3.8)	(0.1–3.5)	(0.1–3.5)	(0.1–3.3)	(0.1–2.8)	

*IQR* interquartile range, *AMH* anti-Mullerian hormone.

^a^*P-*values calculated with the used of chi–square tests for categoric variables and Kruskal–Wallis tests for maternal age.

^b^Sperm parameters are less than WHO2010: volume 1.5 mL, total sperm number 39 million per ejaculate, sperm concentration 15 million per mL, total motility 40%, morphologically normal forms 4.0%.

**Table 2:** Effect of each characteristics on fertilization and high-grade blastocyst rate in IVF and ICSI by multiple regression analysis.

	IVF				ICSI			
	Normal fertilization rate		High-grade blastocyst rate^a^		Normal fertilization rate		High-grade blastocyst rate^a^	
	β	*P-*values	β	*P-*values	β	*P-*values	β	*P*-values
BMI	0.007	0.579	0.008	0.587	− 0.007	0.506	− 0.001	0.908
Maternal age	0.028	0.098	− 0.148	< 0.001	− 0.037	0.010	− 0.206	< 0001
First cycle in oocyte retrieval	0.010	0.881	0.060	< 0.001	0.030	0.014	0.066	< 0.001
History of delivery	0.010	0.438	− 0.007	0.642	0.029	0.007	0.003	0.785
History of miscarriage								
≥ 1	0.002	0.910	0.012	0.455	− 0.004	0.738	0.006	0.659
≥ 2	0.027	0.068	0.020	0.205	0.011	0.325	0.007	0.509
Endometriosis	− 0.003	0.829	0.017	0.239	0.012	0.274	− 0.010	0.364
Male factor infertility^a^	− 0.075	< 0.001	0.001	0.944	− 0.005	0.613	− 0.015	0.179
AMH	− 0.012	0.395	< 0.001	0.979	0.015	0.227	− 0.004	0.758

*IVF* in vitro fertilization, *ICSI* intracytoplasmic sperm injection, *β* standardized partial regression coefficient, BMI: body mass index, AMH: anti-Mullerian hormone.

^a^Gardner Schoolcraft criteria of ≥ 3BB.

^b^Sperm parameters are less than WHO2010: volume 1.5 mL, total sperm number 39 million per ejaculate, sperm concentration 15 million per mL, total motility 40%, morphologically normal forms 4.0%.

The characteristics of the confounding factors in FET are shown in Table 3. Logistic regression analysis in FET showed that maternal age, first cycle in FET, history of delivery, history of miscarriage, and day 5 blastocyst transfer were associated with clinical pregnancy. Meanwhile, maternal age, first cycle in FET, history of delivery, history of recurrent miscarriage, day 5 blastocyst transfer, and uterine factor infertility were associated with miscarriage. Maternal age, first cycle in FET, history of delivery, history of miscarriage, and day 5 blastocyst transfer were associated with live birth (Table 4). The Chi-square test showed no significant difference in the miscarriage rate among the five BMI groups (*P* = 0.193). However, there was a significant difference in the clinical pregnancy and live birth rate (*P* = 0.014, and *P* = 0.001, respectively). Following residual analysis, the clinical pregnancy rate was significantly lower for BMI ≥ 25 than for other groups (adjusted standardized residual (Rad) =  − 2.7). In addition, live birth rates were significantly higher in BMI < 18.5 (Rad = 3.0) and significantly lower in BMI ≥ 25 (Rad =  − 3.1). However, after PS matching, no significant differences were observed (Clinical pregnancy: *P* = 0.313, Miscarriage: *P* = 0.268, Live birth: *P* = 0.578) (Table 5). In clinical pregnancy, the covariates included the first cycle in FET, maternal age, delivery history, and day 5 embryo transfer. After PS matching, 6,022 cycles were enrolled in the analysis. Meanwhile, in miscarriage, the covariates included first cycle in FET, maternal age, delivery history, history of recurrent miscarriage, day 5 blastocyst transfer, and uterine factor infertility. After PS matching, 1,846 cycles were enrolled in the analysis. In the live birth rate, the covariates included the first cycle in FET, maternal age, history of delivery, history of miscarriage, and day 5 blastocyst transfer. After PS matching, 5,428 cycles were enrolled in the analysis.

**Table 3:** Population characteristics in highest-grade embryo transfer^a^.

	Body Mass Index (kg/m^2^)					
	< 18.5	18.5–20.0	20.0–22.5	22.5–25.0	≥ 25.0	

	n = 1154	n = 1852	n = 2645	n = 1092	n = 379	*P-*values^b^
Maternal age, year, median	36.0	36.0	37.0	37.0	38.0	< 0.001
(IQR)	(29.0–43.0)	(30.0–42.0)	(31.0–43.0)	(31.0–43.0)	(32.0–44.0)	
First cycle in FET (%)	547 (47.4)	830 (44.8)	1147 (43.3)	468 (42.8)	154 (40.6)	0.073
History of delivery (%)	372 (32.2)	580 (31.3)	790 (29.8)	304 (27.8)	74 (19.5)	< 0.001
History of miscarriage (%)						
≥ 1	393 (34.0)	687 (37.1)	1063 (40.1)	443 (40.5)	155 (40.9)	0.002
≥ 2	72 (6.2)	212 (11.4)	340 (12.8)	141 (12.9)	42 (11.0)	< 0.001
No. of embryos transferred^c^ (%)						
1	1152 (99.8)	1849 (99.8)	2639 (99.7)	1089 (99.7)	378 (99.7)	0.965
2	2 (0.1)	3 (0.1)	6 (0.2)	3 (0.2)	1 (0.2)	0.965
Day 5 blastocyst transfer^d^ (%)	1024 (88.8)	1572 (84.8)	2261 (85.4)	929 (85.0)	324 (85.4)	0.031
Uterine factor infertility	5 (0.4)	15 (0.8)	10 (0.3)	4 (0.3)	2 (0.5)	0.307

*IQR* interquartile range, *FET* frozen-thawed embryo transfer.

^a^Gardner Schoolcraft criteria of ≥ 3AA.

^b^*P-*values calculated with the used of chi–square tests for categoric variables and Kruskal–Wallis tests for maternal age.

^c^No. of single embryo transfer or double embryo transfer/total No. of FET.

^d^No. of Day 5 embryo transfer/total No. of FET.

**Table 4:** Effect of each characteristics on clinical pregnancy, miscarriage and live birth rate in FET by Logistic regression analysis.

	Clinical pregnancy			Miscarriage			Live birth		
	OR	95% CI	*P-*values	OR	95% CI	*P*-values	OR	95% CI	*P-*values
Maternal age, year, median	0.892	0.881–0.903	< 0.001	1.150	1.126–1.175	< 0.001	0.873	0.862–0.885	< 0.001
First cycle in FET	1.340	1.211–1.483	< 0.001	0.766	0.650–0.903	0.001	1.317	1.184–1.465	< 0.001
History of delivery	1.262	1.132–1.407	< 0.001	0.837	0.705–0.993	0.042	1.290	1.152–1.445	< 0.001
History of miscarriage									
≥ 1	0.592	0.536–0.654	< 0.001	0.983	0.817–1.183	0.857	0.694	0.623–0.773	< 0.001
≥ 2	0.555	0.476–0.647	< 0.001	1.597	1.205–2.116	0.001	0.686	0.564–0.834	< 0.01
Double embryos transfer	1.293	0.448–3.732	0.634	1.129	0.244–5.213	0.877	1.036	0.324–3.316	0.952
Day 5 blastocyst transfer^a^	1.550	1.346–1.785	< 0.001	0.639	0.508–0.804	< 0.001	1.779	1.518–2.085	< 0.001
Uterine factor infertility	1.381	0.688–2.771	0.364	2.812	1.187–6.660	0.019	0.815	0.398–1.667	0.575

*OR* odds ratio, *95% CI* 95% confidence interval, *FET* frozen-thawed embryo transfer.

^a^Day 7 blastocyst 20 cycle were excluded.

**Table 5:** Effect of BMI on clinical pregnancy, miscarriage and live birth rate in FET with highest-grade embryo transfer^a^ after PS matching.

BMI five groups (kg/m^2^)	Original				After PS matching		
	Number	Rate (%)	*P*-values^b^	Rad	Number	Rate (%)	*P-*values^b^
Clinical pregnancy							
< 18.5	1154	56.4	0.014	1.8	943	50.7	0.313
18.5–20.0	1852	55.0		1.0	1566	50.4	
20.0–22.5	2645	52.8		− 1.5	2248	48.8	
22.5–25.0	1092	54.9		0.6	935	52.2	
≥ 25	379	47.2		− 2.7	330	46.9	
Total	7122				6022		
Miscarriage^d^							
< 18.5	648	21.2	0.193		283	48.4	0.268
18.5–20.0	1002	24.6			486	50.6	
20.0–22.5	1381	24.9			670	50.7	
22.5–25.0	590	25.4			323	46.4	
≥ 25	175	29.1			84	59.5	
Total	3796^c^				1846		
Live birth^e^							
< 18.5	1153	44.2	0.001	3.0	918	51.7	0.578
18.5–20.0	1847	40.8		0.6	1440	49.5	
20.0–22.5	2640	39.2		− 1.5	2010	49.0	
22.5–25.0	1087	40.4		0.1	815	51.5	
≥ 25	378	32.8		− 3.1	245	48.9	
Total	7105^c^				5428		

*BMI* body mass index, *FET* frozen-thawed embryo transfer, *PS* propensity score, *Rad* adjusted standardized residual.

^a^Gardner Schoolcraft criteria of ≥ 3AA.

^b^*P*-values calculated with the used of chi-square tests. If there were significant differences, residual analysis was then performed.

^c^Seventeen cycles with unknown outcome after pregnancy were excluded.

^d^Including stillbirth.

^e^Live birth rate was calculated from the number of live births/number of patients.

## Discussion

This study identified no significant differences between the five BMI groups for normal fertilization, high-grade blastocyst, clinical pregnancy, live birth, and miscarriage rate in FET. Therefore, BMI may not adequately predict ART outcomes. According to a previous report, endocrine changes related to obesity, such as hyperandrogenism and insulin resistance, and alterations in local insulin-like growth factors, cytokines, adiponectin, and leptin levels, may play a major role in the adverse effects of increased BMI on ART outcomes ^10^. However, based on our results, these factors may act independently of BMI.

Previous reports have shown that the impact of BMI on ART outcomes remains controversial. For all fresh autologous IVF cycles in the United States between 2008 and 2013, Kawwass et al. reported that underweight women (BMI < 18.5), compared with normal-weight women (BMI 18.5–24.9), had a statistically significant decrease in the chance of intrauterine pregnancy (95% CI 0.96–0.99) and live birth (95% CI 0.93–0.98) per transfer. Women with obesity (BMI ≥ 30.0) had a statistically decreased likelihood of intrauterine pregnancy and live birth (95% CI 0.94–0.95; 95% CI 0.86–0.88, respectively). In addition, obesity was associated with a statistically significant increase in miscarriage risk (95% CI 1.20–1.26) ^8^. Similarly, Nogales et al. ^15^ and Zhang et al. ^21^ reported a significant relationship between BMI and miscarriage rates. Meanwhile, Kudesia et al. conducted a retrospective cohort study that included 51,198 women who initiated their first-fresh autologous IVF or ICSI cycle at 13 fertility centers in the USA between 2009 and 2015 ^11^. They reported that a BMI above the normal range was an independent negative prognostic factor for fresh embryo transfer in cycle cancellation, oocyte and embryo counts, and ongoing clinical pregnancy.

The studies by Kawwass et al. and Kudesia et al. utilized only fresh embryo transfers. In addition, in the reports from Nogales et al. there is a possibility that the results may be affected by the procedures and culture methods between institutions because they were conducted at multiple centers. In the reports from Zhang et al., most embryos were at the cleavage stage, not blastocyst. Therefore, there were many previous reports of the effects of BMI on ART outcomes have included fresh embryo transfers and Western populations, but not FET and Asians. In addition, many previous studies have not distinguished day 5 and day 6 blastocysts, which could impact the evaluation of ART outcomes. Furthermore, most reports on preimplantation genetic testing for aneuploidies and donor oocytes have not investigated their effects on embryogenesis. In contrast, the sample size in the present study was large, and the ART method and procedure were uniform because the study was conducted at a single institution. Our study reported on FET, a mainstream procedure in Japan ^22^. Only the highest-grade embryos, such as 3AA or higher, were analyzed in this study. In addition, we considered the effect on ART outcomes by distinguishing day 5 from day 6 embryos. In many previous reports, BMI was divided into three or four groups; however, this study subdivided BMI groups further based on the World Health Organization guidelines. We believe this method has enabled us to investigate the effect of BMI on ART and pregnancy outcomes more accurately, thus benefiting East Asians who reportedly have a lower BMI than Western populations ^1^. This study had two limitations. First, there may have been a loss in statistical power due to the division of BMI classification into more groups than in previous studies; any minor effects of BMI may have therefore been missed. Second, relatively few cases had a BMI ≥ 30, making it difficult to evaluate ART outcomes in patients with high BMI. Despite these limitations, this study provides insight into the effect of BMI on ART and pregnancy outcomes.

In this study, BMI did not affect ART outcomes; however, we found that it was associated with the risk of hypertensive disorders, diabetes, and complications in offspring in a previous study. Therefore, we continue to recommend that women with obesity be required to lose weight before ART. However, weight loss does not appear to affect ART outcomes based on the results of this study and previous studies. In addition, our results indicate that physicians should not delay infertility treatment in older adult women. In the future, we believe it is necessary to increase the number of low and high BMI (< 15 and ≥ 30) cases and investigate them to improve fertility outcomes further.

## Data Availability

The datasets analyzed during the current study are available from the corresponding author on reasonable request.

## References

[1] NCD Risk Factor Collaboration (NCD-RisC) (2021). Heterogeneous contributions of change in population distribution of body mass index to change in obesity and underweight. Elife.

[2] Bartolacci A (2019). Maternal body mass index affects embryo morphokinetics: A time-lapse study. J. Assist. Reprod. Genet..

[3] Bellver J, Brandão P, Alegre L, Meseguer M (2021). Blastocyst formation is similar in obese and normal weight women: A morphokinetic study. Hum Reprod..

[4] Stovezky YR, Romanski PA, Bortoletto P, Spandorfer SD (2021). Body mass index is not associated with embryo ploidy in patients undergoing in vitro fertilization with preimplantation genetic testing. Fertil. Steril..

[5] Hughes LM, McQueen DB, Jungheim ES, Merrion K, Boots CE (2022). Maternal body mass index is not associated with increased rates of maternal embryonic aneuploidy. Fertil. Steril..

[6] Cozzolino M, García-Velasco JA, Meseguer M, Pellicer A, Bellver J (2021). Female obesity increases the risk of miscarriage of euploid embryos. Fertil. Steril..

[7] Meiting Qiu Yu, Tao YK, Wang Y (2019). Effect of body mass index on pregnancy outcomes with the freeze-all strategy in women with polycystic ovarian syndrome. Fertil. Steril..

[8] Kawwass JF (2016). Extremities of body mass index and their association with pregnancy outcomes in women undergoing in vitro fertilization in the United States. Fertil. Steril..

[9] Provost MP (2016). Pregnancy outcomes decline with increasing body mass index: Analysis of 239,127 fresh autologous in vitro fertilization cycles from the 2008–2010 Society for Assisted Reproductive Technology registry. Fertil. Steril..

[10] Arabipoor A, Ashrafi M, Hemat M, Zolfaghari Z (2019). The effects of maternal and paternal body mass index on live birth rate after intracytoplasmic sperm injection cycles. Int. J. Fertil. Steril..

[11] Kudesia R (2018). The effect of female body mass index on in vitro fertilization cycle outcomes: A multi-center analysis. J. Assist. Reprod. Genet..

[12] Prost E, Reignier A, Leperlier F (2020). Female obesity does not impact live birth rate after frozen-thawed blastocyst transfer. Hum. Reprod..

[13] Lan L (2017). Systematic review and meta-analysis of the impact of preconception lifestyle interventions on fertility, obstetric, fetal, anthropometric and metabolic outcomes in men and women. Hum Reprod..

[14] Insogna IG, Lee MS, Reimers RM, Toth TL (2017). Neutral effect of body mass index on implantation rate after frozen-thawed blastocyst transfer. Fertil. Steril..

[15] del armenNogales M (2021). Association between clinical and IVF laboratory parameters and miscarriage after single euploid embryo transfers. Reprod. Biol. Endocrinol..

[16] van Dammen L (2021). A lifestyle intervention randomized controlled trial in obese women with infertility improved body composition among those who experienced childhood adversity. Stress Health..

[17] Yang W (2018). Body mass index and basal androstenedione are independent risk factors for miscarriage in polycystic ovary syndrome. Reprod. Biol. Endocrinol..

[18] Xiong Y-Q (2020). Association between prepregnancy subnormal body weight and obstetrical outcomes after autologous in vitro fertilization cycles: Systematic review and meta-analysis. Fertil. Steril..

[19] Gardner DK, Lane M, Stevens J, Schlenker T, Schoolcraft WB (2000). Blastocyst score affects implantation and pregnancy outcome; towards a single blastocyst transfer. Fertil. Steril..

[20] Cooper TG (2010). World Health Organization reference values for human semen characteristics. Hum. Reprod. Updat..

[21] Zhang J (2019). Effect of body mass index on pregnancy outcomes in a freeze-all policy: An analysis of 22,043 first autologous frozen-thawed embryo transfer cycles in China. BMC Med..

[22] Ishihara O (2021). Assisted reproductive technology in Japan: A summary report for 2018 by the Ethics Committee of the Japan Society of Obstetrics and Gynecology. Reprod. Med. Biol..

